# Supplementary figures and images for: Soil aggregates indirectly influence litter carbon storage and release through soil pH in the highly alkaline soils of north China
Source: PeerJ. 2019 Oct 29;7:e7949. doi: 10.7717/peerj.7949 (PMC6824446; doi:10.7717/peerj.7949)

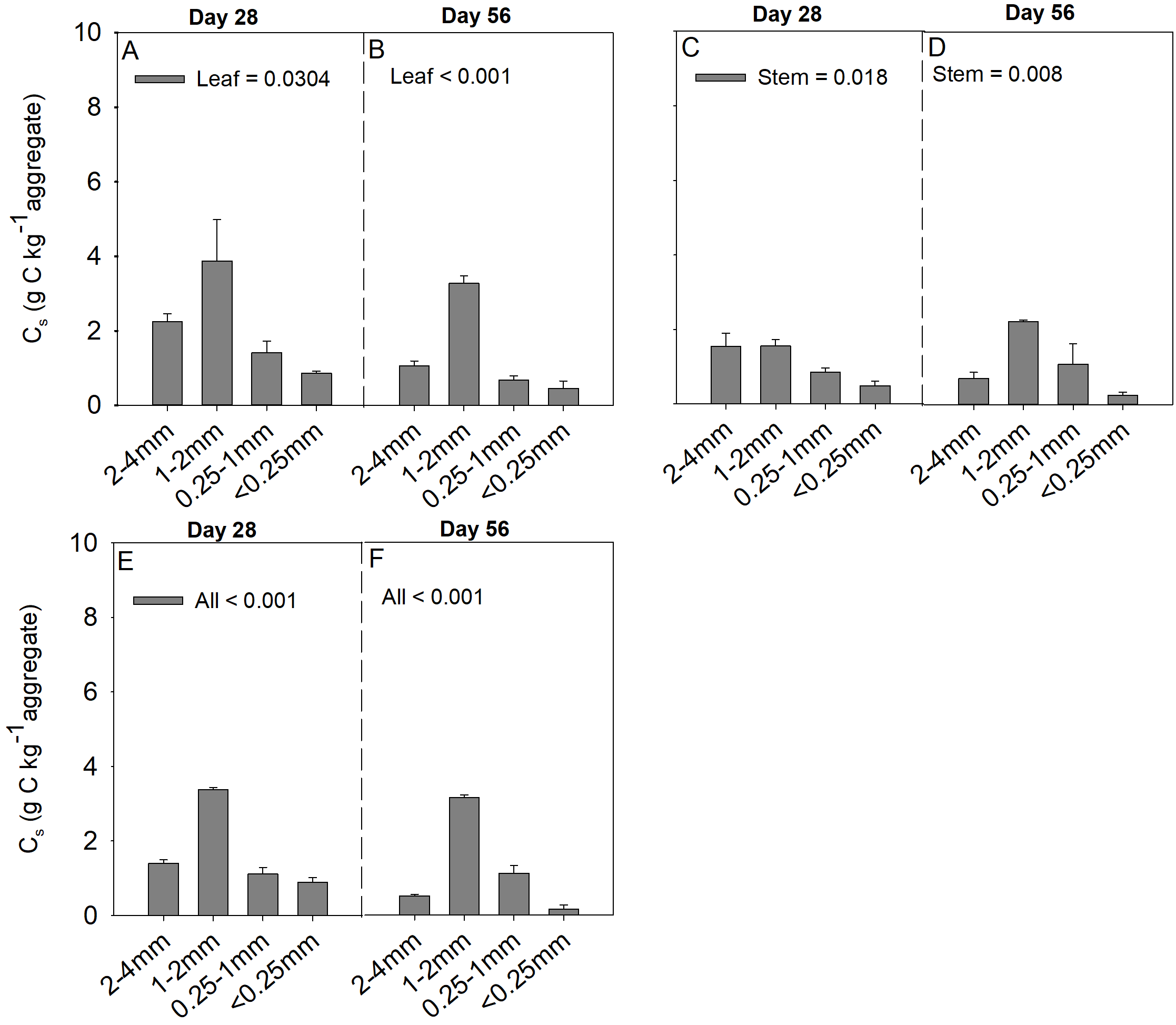

Supplement: Supplemental Information 2 — The error bars show the SE of the means for n = 3. [file peerj-07-7949-s002.png]

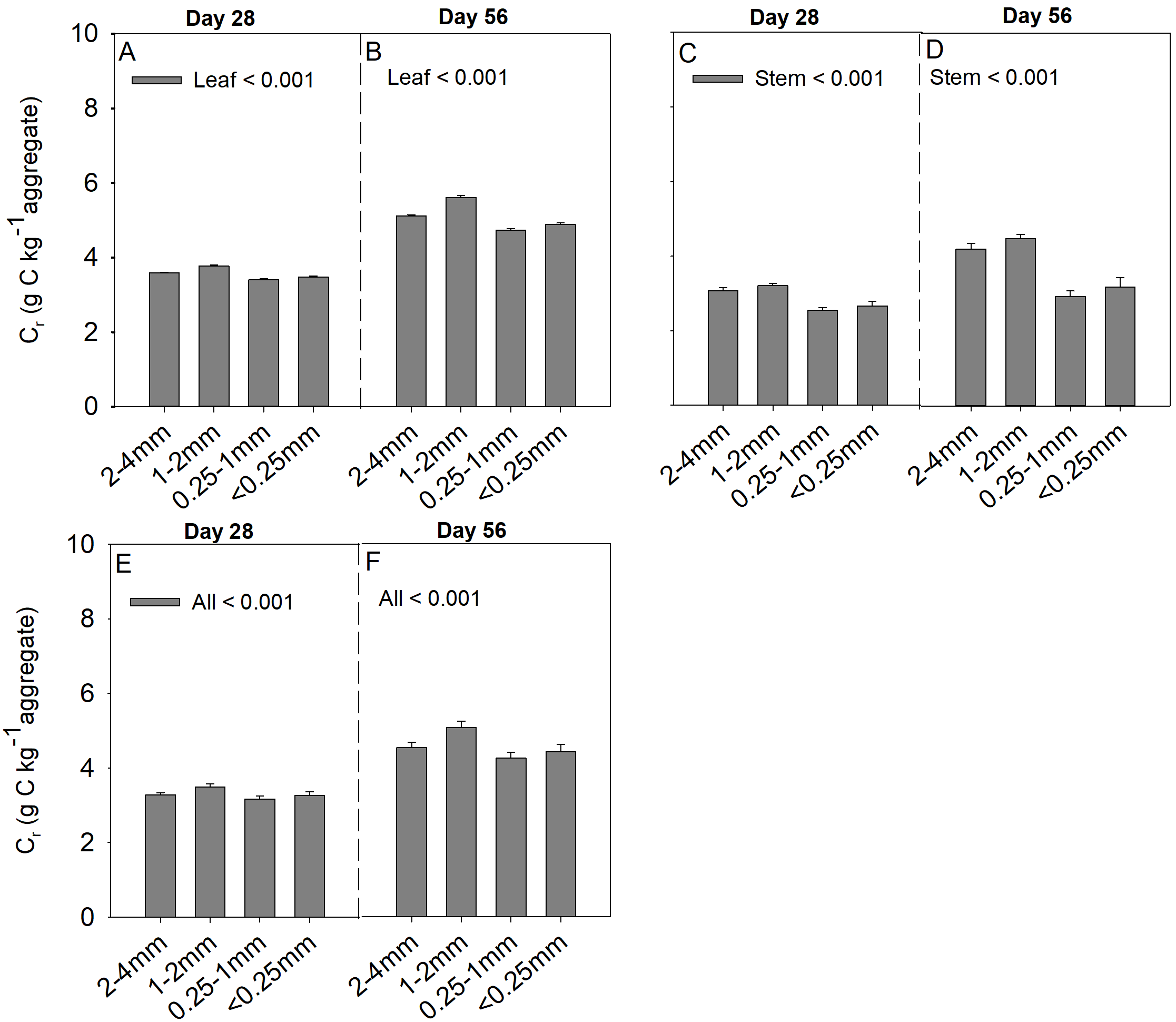

Supplement: Supplemental Information 3 — The error bars show the SE of the means for n = 3. [file peerj-07-7949-s003.png]

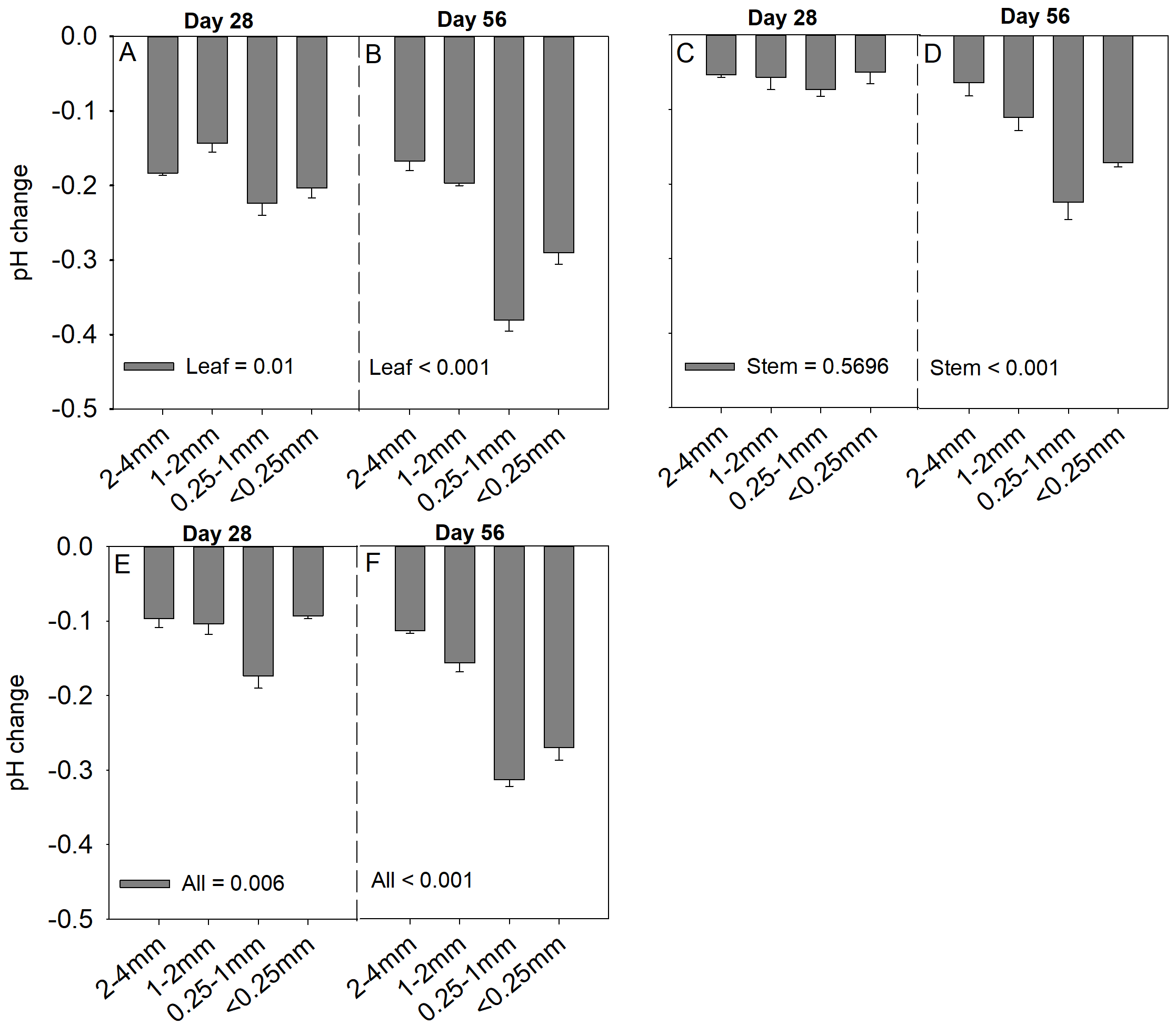

Supplement: Supplemental Information 4 — The error bars show the SE of the means for n = 3. [file peerj-07-7949-s004.png]
